# Efficacy of Esthetic Retainers: Clinical Comparison between Multistranded Wires and Direct-Bond Glass Fiber-Reinforced Composite Splints

**DOI:** 10.1155/2011/548356

**Published:** 2011-10-27

**Authors:** Andrea Scribante, Maria Francesca Sfondrini, Simona Broggini, Marina D'Allocco, Paola Gandini

**Affiliations:** ^1^Department of Orthodontics and Department of Surgical Sciences, University of Pavia, Piazzale Golgi 2, 27100 Pavia, Italy; ^2^Department of Orthodontics, University of Pavia, Piazzale Golgi 2, 27100 Pavia, Italy

## Abstract

The purpose of this longitudinal prospective randomized study was to evaluate the reliability of two different types of orthodontic retainers in clinical use: a multistrand stainless steel wire and a polyethylene ribbon-reinforced resin composite. Moreover the level of satisfaction of the patient about the esthetic result was also analyzed by means of a Visual Analogue Scale (VAS). 34 patients (9 boys and 25 girls, mean age 14.3), in the finishing phase of orthodontic treatment, were selected for the study. Since splints were applied the number, cause, and date of splint failures were recorded for each single tooth over 12 months. Statistical analysis was performed using a paired *t*-test, Kaplan Meier survival estimates, and the log-rank test. Kruskal Wallis test was performed to analyze VAS recordings. Differences between the bond failure rates were not statistically significant. Esthetic result of VAS was significantly higher for polyethylene ribbon-reinforced resin retainers than for stainless steel wires.

## 1. Introduction

Bonded lingual retainers are used principally for long-term retention of treated orthodontic cases and for the permanent splinting of periodontally involved teeth [1]. 

The duration of retention should be decided for each case specifically in conjunction with the patient [2] taking into consideration future growth [3].

Many appliance types have been used for the retention of posttreatment tooth position [4].

Fixed lingual or palatal retainers have significant advantages for patient comfort and esthetic acceptability. They can be placed directly [5, 6] or indirectly [7]. The placement of a bonded retainer is technique sensitive [8, 9].

Spiral or multistrand wires appear to be the most popular for direct-bonded retainers [10].

The main advantage of the use of multistrand wire is the irregular surface that offers increased mechanical retention for the composite without the need for the placement of retentive loops [11]. Moreover another asset is the flexibility of the wire that allows physiologic movement of the teeth, even when several adjacent teeth are bonded [12].

Bonded retainers appear to be well accepted by patients and are relatively independent of patient cooperation [13].

 Although traditional methods are successful, splinting teeth with reinforcement fibers that can be embedded in composites has gained popularity in last years [14].

Fiber-reinforced composite (FRC) containing various fibers such as carbon, polyaramid, polyethylene, and glass has received increasing acceptance as restorative materials [15].

Reinforced polyethylene fiber material was successfully used for fixed orthodontic retainers [16], space maintainers [17], temporary postorthodontic fixation devices to attach a pontic to abutment teeth during periodontal therapy [18, 19], and posttraumatic stabilization splints [20].

The polyethylene fiber material adapted easily to dental contours and could be manipulated during the bonding process. It also has acceptable strength because of integration of fibers with composite resin that leads to good clinical longevity [14].

The bonded orthodontic splint provides the patient with an efficient esthetic retainer that can be maintained in the long term [21]. The most common failure type is detachment at the wire-composite interface because of insufficient adhesive over the wire or unfavorable occlusal contacts, which results in abrasion of the composite [5, 22]. The abrasion of mandibular retainers has been attributed to mechanical forces such as toothbrushing and chewing [23].

When clinical failure of bonded orthodontic retainers is observed relapse can occur [24]. Therefore the purpose of this clinical study was to evaluate the reliability of two different orthodontic retainers: multistrand stainless steel wire and polyethylene fiber-reinforced resin composite (FRC). It was also analyzed the level of satisfaction of the patient about the esthetic result such as visibility of the retainer while talking and smiling, by means of VAS (Visual Analogue Scale).

The null hypothesis of the study was that there is no statistically difference in bond failure rate and VAS records between the two different retainers. 

## 2. Materials and Method

A total of 34 patients (mean age: 14.3) in the finishing phase of orthodontic treatment, attending the Orthodontic Department “S. Palazzi” of Pavia University, were selected. For this randomized clinical study the inclusion criteria were correct dental alignment, need for permanent orthodontic retention in the lower anterior segments, and being free of occlusal interferences in order to eliminate the influence of trauma. Institutional approval was achieved from the department. Written patient and parental informed consents were obtained.

**Figure 1:**
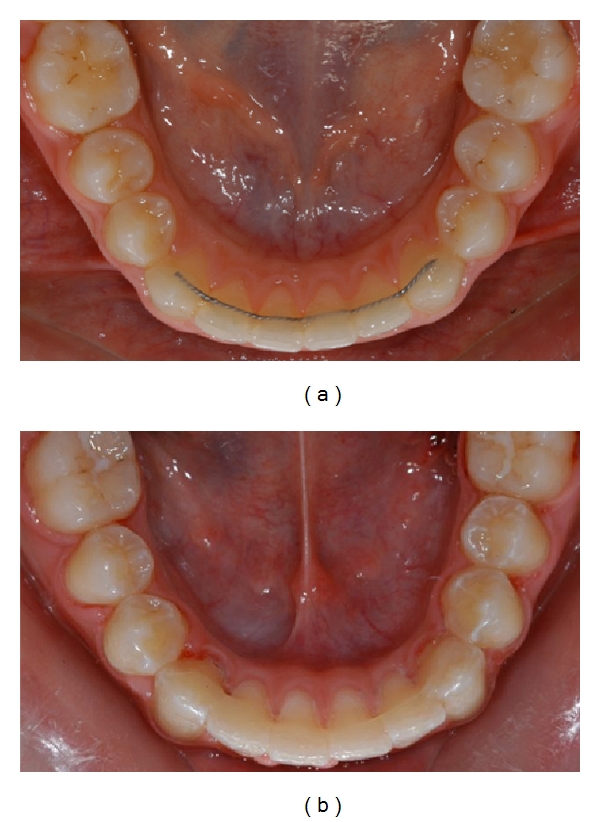
Metallic (a) and FRC (b) retentions.

Patients were randomly assigned to one of two groups. In the first group was applied a multistrand stainless steel wire (Ortosmail Krugg, Milan, Italy), as showed in Figure 1(a) and in the second one a polyethylene fiber-reinforced resin composite (InFibra TPItalia, Gorle, Italy), as showed in Figure 1(b). The retainers were applied in the mandibular arch from right to left canine. Since bonding of the retainer all patients were followed with periodic monitoring visits at 30, 60, 120, 180, 360 days to evaluate detachments. 

 Specific data were collected: date of bonding, type of splint, position, number of teeth involved, detachment (position and number), followup at 30, 60, 120, 180, 360 days.

34 patients were enroled: 17 with a multistrand wire retainer (total number of teeth bonded 102), while 15 with a polyethylene fiber-reinforced resin composite (total number of teeth bonded 90). 

0.0175′′ multistranded wire and polyethylene fiber reinforced resin preimpregnated with Transbond XT Primer (3M Unitek, Monrovia, Calif, USA) were used. 

All teeth were isolated with cheek retractors and cleaned with a mixture of water and fluoride-free pumice using a rubber polishing cup in a low-speed handpiece. The teeth were rinsed up with water and dried with an oil-free syringe. After lingual surfaces cleaning, each tooth was etched with 37% orthophosphoric acid gel (3M Unitek, Monrovia, Calif, USA) for 20 seconds and then rinsed and air-dried. The retainers were accurately located on the lingual surface and a thin layer of bonding Transbond XT Primer (3M Unitek, Monrovia, Calif, USA) was applied and then light cured with a halogen curing unit (Opitlux 501; SDS Kerr, Danbury, Conn; light intensity, 930 mW/cm^2^; wavelength range, 400–505 nm) for 20 seconds, as suggested by the manufacturer. 

A small amount of Trasbond XT Resin (3M Unitek, Monrovia, Calif, USA) was placed to cover both metallic and FRC splints and light-cured for 40 seconds, as suggested by the manufacturer. Finishing was conducted using diamond burs and polishing discs. 

Each patient was asked to express an opinion about the esthetic result such as visibility of the retainer while talking and smiling by means of VAS (Visual Analogue Scale) in which the 0 point means poor esthetic result effect and 10 means excellent esthetic effect.

Statistical analysis was performed with a computer software (Stata 7, College Station, Tex). Paired *t*-test was applied to determine differences in detachment existed among the groups. In addition to the simple event of failure, the time of bonding failure was also considered. Kaplan-Meier estimates of survival plots were constructed, and a log-rank test was carried out to compare the distribution of survival times in the two groups. 

FRCs and stainless steel splints' VAS was analyzed by means of Kruskal Wallis test.

Significance for all statistical tests was predetermined at *P* < 0.05.

## 3. Results

As reported in Table 1, no significant differences in total number of detachments between the two splints were detected (*P* > 0.05). At the end of the followup the percentage of total detachment of the two different retainers were 22,54% for multistrand stainless steel wire and 14,45% for polyethylene ribbon-reinforced resin retainer, respectively. 

**Figure 2:**
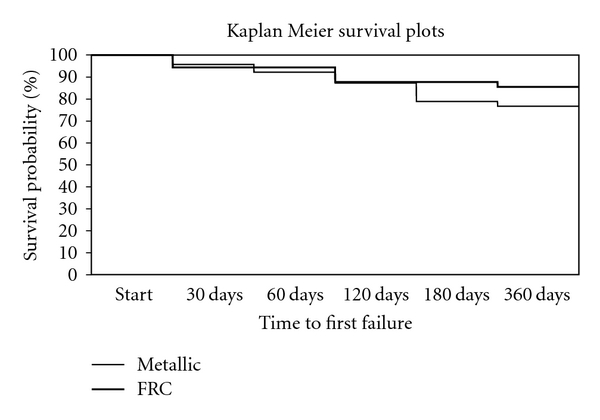
Survival plots for multistrand wire and FRC reteiners.

Kaplan-Meier survival plots for the two types of retainers are shown in Figure 2. There was no statistically significant difference in retainers failure risk over the 12 months of followup (Hazard Ratio: 0.77; Confidence Interval 95%: 0.31–1.93; log-rank test: *P* = 0.58).

During the 12-month retention period no wire or FRC fractures were found in both groups. Moreover no wear of the FRC surface was detected.

Table 2 shows visual analogue scale (VAS) of the level of satisfaction of the patient about the esthetic result. Significant differences were reported between the two groups (*P* = 0.026). The visual analogue scale (VAS), in which “0” point means very poor esthetic effect and “10” means excellent esthetic effect, showed that patients with multistranded stainless steel wire expressed a mean value of satisfaction of 8.24, whereas patients with polyethylene fiber reinforced resin retainer for lingual retention expressed a mean value of satisfaction of 9.73. 

## 4. Discussion

The null hypothesis of the study was partially rejected. No significant difference was detected between the percentage of failures of the two types of bonded retainers whereas significant differences in VAS records were reported. 

Survival plots show that polyethylene fiber reinforced resin retainer had a survival rate lower than the multistrand stainless steel wire after 30 days. At the subsequent followups, especially at 180 and 360 days, FRCs present a survival plot that overtake multistrand stainless steel wires. Neverthless the difference between the two groups was not significant (*P* = 0.58). FRC retainers have good mechanical properties and are clinically reliable for long term [25]. Reinforcement of polymers with long, continuous fibers is effective for many applications [26]. 

A previous investigation evaluated different orthodontic adhesives for FRC bonding and Transbond XT exhibited higher shear bond strength values than both flow composite and glass ionomer cement [27]. Therefore in the present investigation Trasbond XT Primer and Trasbond XT Resin (3M Unitek, Monrovia, California, USA) were used. There are few clinical investigations that have compared failure rate of multistrand wire retainer with a polyethylene fiber reinforced resin retainer. The reliability of posttreatment canine-to-canine retention with resin composite retainers reinforced with plasma-treated woven polyethylene ribbons was compared to the reliability of directly bonded, multistranded wire retainers by Rose et al. [16]. This prospective study, based on an assessment of 20 patients, demonstrated that ribbon-reinforced retainer remained in place for shorter period of time than multistranded wire. Similar findings have been described for glass fiber reinforced retainers when compared with multistranded retainers [28]. These are in disagreement with present study, which shows no statistically significant differences between the two types of splint for the percentage of survival after 12 months. The reason of the different results could be ascribed to the use of different materials and different bonding techniques in the studies.

Zachrisson in a study of 1977 [5] evaluated 43 direct-bonded mandibular canine-to-canine retainers after observation period between 12 to 30 months. Results indicate that the bonded retainer has all the advantages of a fixed soldered retainer, in addition to being invisible. The failure rate in terms of loose retainers (11,6%) was similar to that of the present investigation. 

Moreover even with higher flexibility of polyethylene FRC versus glass FRC in terms of material properties and diameter, some limitations in the clinical use of polyethylene FRCs still persist. In fact in marginal areas the fibers may become exposed and come into contact with oral tissues, saliva, and microbes. Polyethylene FRCs were found to be significantly rougher than glass FRCs and restorative materials. This roughness can result in a higher retention of bacteria than the other materials tested [29–31].

The reinforcement of polymers with a ribbon layer slightly increases the transverse strength, but the adherence of the polyethylene fibers to the base polymer have been shown to be poor [32], and this could represent another limitation for long-term stability of polyethylene FRCs.

Finally in the present investigation a visual analogue scale (VAS) was used for the subjective evaluation to assess the level of patient satisfaction regarding the esthetic outcome of the metallic and FRC splints. This kind of investigation was used in the literature specially to evaluate esthetic result after prosthodontic treatment instead is not widely used in orthodontics [33–35].

In the present investigation significant differences in VAS records were reported between the two groups. In fact esthetic result of FRC retainers was significantly higher than stainless steel multistranded wire.

## 5. Conclusions

The results showed that there was no statistically significant difference between the failure rate of the two types of bonded retainers.

VAS records showed that esthetic result was significantly higher for polyethylene ribbon-reinforced resin retainers.

## Figures and Tables

**Table 1:** Distribution of bond failures for the different retainer types. Numbers of single tooth detachment.

	No. of bonded	No. of failed	Percentage
Stainless steel wire	102	23	22.54%
FRC	90	13	14.45%
Total	192	36	18.75%
Paired *t*-test			ns

**Table 2:** Descriptive statistics of VAS values.

	Mean	Std. deviation	Minimum	Median	Maximum
Stainless steel wire	8.24	1.39	4.50	8.50	10.00
FRC	9.73	0.42	9.00	10.00	10.00
Paired *t*-test	0.026				
